# Structure of the native chemotaxis core signaling unit from phage E-protein lysed *E. coli* cells

**DOI:** 10.1128/mbio.00793-23

**Published:** 2023-09-29

**Authors:** C. Keith Cassidy, Zhuan Qin, Thomas Frosio, Khoosheh Gosink, Zhengyi Yang, Mark S. P. Sansom, Phillip J. Stansfeld, John S. Parkinson, Peijun Zhang

**Affiliations:** 1 Diamond Light Source, Didcot, United Kingdom; 2 Department of Physics and Astronomy, University of Missouri-Columbia, Columbia, Missouri, USA; 3 Division of Structural Biology, Wellcome Trust Centre for Human Genetics, University of Oxford, Oxford, United Kingdom; 4 School of Biological Sciences, University of Utah, Salt Lake City, Utah, USA; 5 Department of Biochemistry, University of Oxford, Oxford, United Kingdom; 6 School of Life Sciences, University of Warwick, Coventry, United Kingdom; 7 Chinese Academy of Medical Sciences Oxford Institute, University of Oxford, Oxford, United Kingdom; University of Washington, Seattle, Washington, USA

**Keywords:** chemotaxis, cryoET, sub-tomogram averaging, *in situ*, cryoEM, chemosensory, phage lysis, MD simulation

## Abstract

Motile bacteria employ conserved chemotaxis networks to detect chemical gradients in their surroundings and effectively regulate their locomotion, enabling the location of essential nutrients and other important biological niches. The sensory apparatus of the chemotaxis pathway is an array of core-signaling units (CSUs) composed of transmembrane chemoreceptors, the histidine kinase CheA and an adaptor protein, CheW. Although chemotaxis pathways represent the best understood signaling systems, a detailed mechanistic understanding of signal transduction has been hindered by the lack of a complete structural picture of the CSU and extended array. In this study, we present the structure of the complete CSU from phage φX174 E protein lysed *Escherichia coli* cells, determined using cryo-electron tomography and sub-tomogram averaging to 12-Å resolution. Using AlphaFold2, we further predict the atomic structures of the CSU’s constituent proteins as well as key protein-protein interfaces, enabling the assembly an all-atom CSU model, which we conformationally refine using our cryo-electron tomography map. Molecular dynamics simulations of the resulting model provide new insight into the periplasmic organization of the complex, including novel interactions between neighboring receptor ligand-binding domains. Our results further elucidate previously unresolved interactions between individual CheA domains, including an anti-parallel P1 dimer and non-productive binding mode between P1 and P4, enhancing our understanding of the structural mechanisms underlying CheA signaling and regulation.

**IMPORTANCE:**

Bacterial chemotaxis is a ubiquitous behavior that enables cell movement toward or away from specific chemicals. It serves as an important model for understanding cell sensory signal transduction and motility. Characterization of the molecular mechanisms underlying chemotaxis is of fundamental interest and requires a high-resolution structural picture of the sensing machinery, the chemosensory array. In this study, we combine cryo-electron tomography and molecular simulation to present the complete structure of the core signaling unit, the basic building block of chemosensory arrays, from *Escherichia coli*. Our results provide new insight into previously poorly-resolved regions of the complex and offer a structural basis for designing new experiments to test mechanistic hypotheses.

## INTRODUCTION

Motile bacteria detect ambient chemical gradients and control locomotion via conserved chemotaxis signaling networks, which enable them to seek out nutrients, potential hosts, and other important biological niches (1). The chemotaxis network of *Escherichia coli* has served for decades as a model system for the study of sensory signal transduction and motility behaviors (2). In this system, thousands of copies of transmembrane (TM) chemoreceptors, the histidine autokinase CheA and adaptor protein CheW, form highly ordered structures known as chemosensory arrays (3), which cooperatively integrate the sensory inputs of multiple receptors to regulate the autophosphorylation activity of CheA. CheA, in turn, donates its phosphoryl groups to the diffusible intracellular response regulator CheY to modulate the cell’s flagellar motors.

Core signaling units (CSU) of the chemosensory array contain six receptor dimers, organized as two trimers of dimers (TODs), a single CheA dimer and two CheW adaptor proteins. The CSU is the minimal molecular unit needed to couple CheA autophosphorylation to receptor control (4). The constituent proteins of CSUs have complex, modular structures. *E. coli* has four canonical chemoreceptors (Tsr, Tar, Trg, and Tap), known as methyl-accepting chemotaxis proteins (MCPs), that form homodimers of largely helical protomers. MCP molecules contain a periplasmic ligand-binding domain (LBD), TM four-helix bundle, and cytoplasmic kinase control domain (2). The cytoplasmic domain itself has a membrane-proximal HAMP (*h*istidine kinases, *a*denylyl cyclases, *M*CP and some *p*hosphatases) domain coupled to a long coiled-coil four-helix bundle, the membrane-distal end of which contains the contact surfaces for TOD formation and CSU assembly.

CheA functions as a homodimer in the CSU; each protomer comprises five domains (P1–P5) joined by flexible linkers (5). The P3, P4, and P5 domains form a compact core through the principal dimerization determinant P3. That dimeric core is integrated into the larger CSU via P5 interactions with the receptor tips and CheW. The P4 domain binds ATP and catalyzes autophosphorylation at a histidine residue in the P1 domain. The P1 and P2 domains are attached to the P3-P4-P5 core via long, disordered linkers and mediate phosphoryl group transfer to CheY. The monomeric CheW protein has a fold homologous to the CheA.P5 domain and interacts with P5 to couple CheA to the receptor trimers. We note that two additional CheW molecules can associate with the periphery of the CSU and contribute to the formation of hexameric CheW rings in the larger array (6 – 8). Those CheW molecules are not essential for kinase regulation in the CSU.

High-resolution CSU structures are central to understanding their molecular signaling mechanisms, particularly receptor-mediated kinase control. Toward this end, cryo-electron tomography (cryoET) has played an essential role in revealing that chemoreceptors in diverse microbial species form TODs organized into a hexagonal lattice with identical spacing (9, 10). In *E. coli*, this hexagonal packing was shown to extend to the CheA/CheW baseplate region at the receptor tip, where rings containing CheA.P5 and CheW were seen to inter-lock adjacent CSUs (6, 7, 11). CryoET studies employing sub-tomogram averaging (STA) have produced increasingly detailed structures of the CSU in thinner array samples, including those of lysed cells (12), minicells (13), and *in vitro* reconstituted arrays (7, 14). Such cryoET density maps have been combined with integrative modeling and molecular simulation techniques to achieve residue-level characterization of CSU structure and dynamics (7, 13, 14). Nevertheless, shortcomings remain: reconstructions from lysed cells have so far achieved only modest resolutions (>20 Å), and minicells can still be relatively thick (~400 nm), which limits the maximum resolution possible. Moreover, although reconstructions of monolayer arrays reached 8-Å resolution, they were formed using soluble receptor molecules that lacked the periplasmic and transmembrane regions.

Here we utilized controlled lysis triggered by induction of a phage E gene to create thin *E. coli* ghost cells containing wild-type chemosensory arrays. We further used STA to obtain a density map of the complete CSU and AlphaFold2 to produce full-length atomic models of the CSU proteins that could be flexibly fitted to the cryoET density map. All-atom molecular dynamics (MD) simulations of the resulting complete CSU model provided new insights into the periplasmic organization of the CSU as well as previously unresolved interactions between individual CheA domains, suggesting a new generation of structure-function experiments to elucidate the workings of chemosensory arrays.

## RESULTS

### Imaging native chemosensory arrays in wild-type *E. coli* ghosts

**FIG 1:**
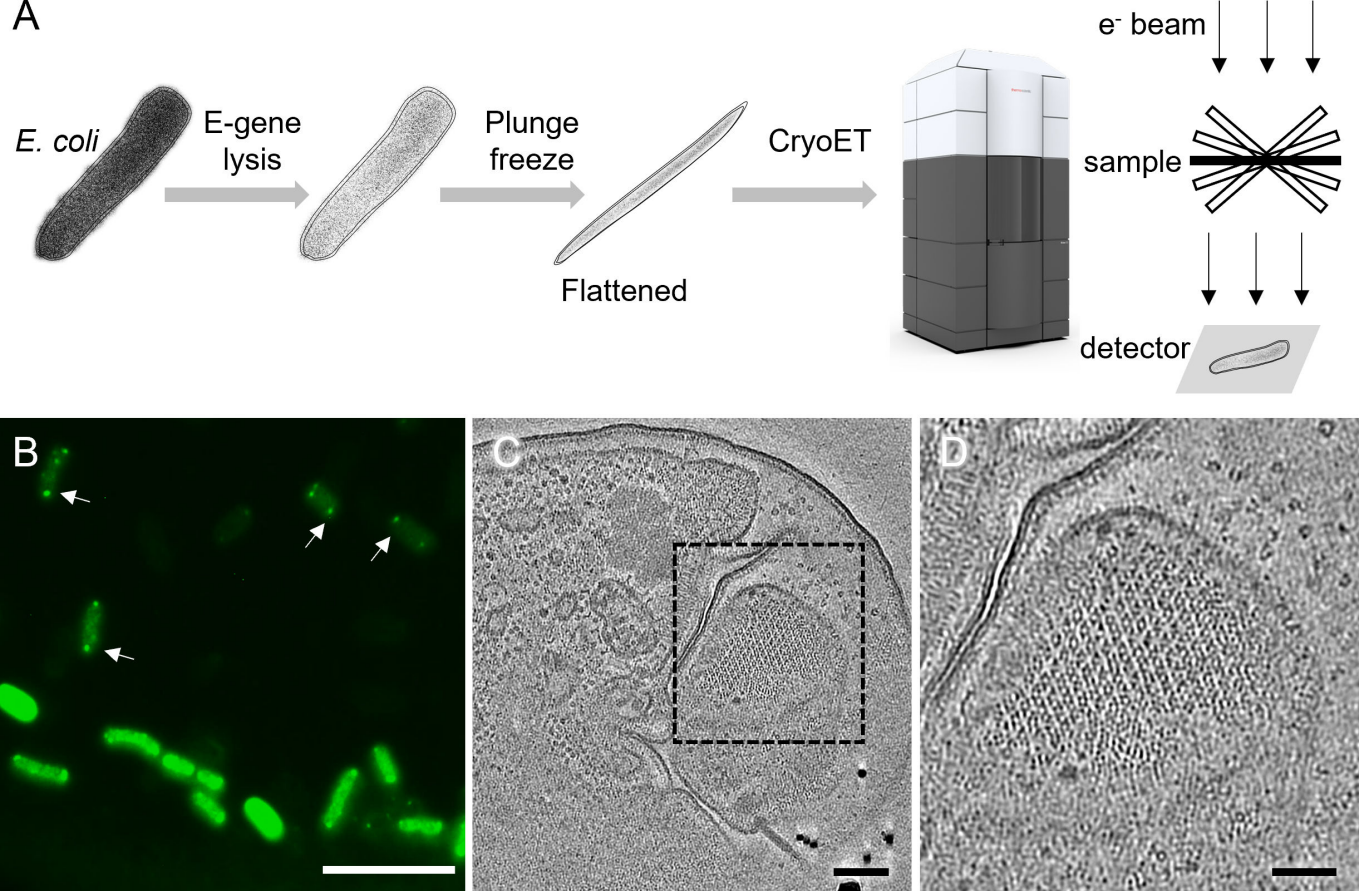
CryoET of native chemosensory arrays in wild-type *E. coli* ghost cells. (**A**) A schematic diagram of the cryoET workflow used in this study. (**B**) A fluorescent light microscopy image of GFP-labeled CheY highlights chemosensory arrays (arrows) near the poles of both E-lysed cells (transparent green) and unlysed cells (bright green). (**C**) A tomographic slice of an E-lysed *E. coli* cell highlighting a patch of the chemosensory array lattice (dashed box). (**D**) A zoom-in view of the area marked by the dashed box in panel C. Scale bars, 10 µm in panel B, 100 nm in panel C, and 50 nm in panel D.

The thickness of native *E. coli* cells (~1.5 µm) prohibits acquisition of quality cryo-tomograms for high resolution structural analysis of chemosensory arrays. We previously developed a method to produce *E. coli* ‘ghost’ cells just before plunge-freezing (15). Upon induction of lysis gene E from the φX174 bacteriophage, spot lesions form in the cell membranes that release the cellular contents while maintaining the overall integrity of the cell envelope. Vitrification of such samples produces thin, flattened cell ghosts (<200 nm) (Fig. 1A). In light microscopy, the *E. coli* ghosts were easily distinguished from unlysed cells due to minimal cytoplasmic green fluorescent protein (GFP)-labeled CheY and had distinct chemosensory arrays as visualized via associated CheY (16) (Fig. 1B, arrows). The *E. coli* ghosts were also easy to identify in cryoEM projection images as they appeared semi-transparent. The resulting reconstructed tomograms display well-ordered hexagonal polar arrays about 200–300 nm across with a 12-nm lattice spacing (Fig. 1C and D; Movie S1).

### CryoET STA structure of the complete CSU in *E. coli* ghosts

CryoET data were collected from the E protein lysed *E. coli* ghosts. The data collection and processing statistics are summarized in Table S1, and the cryoET STA process is illustrated in a workflow schematic (Fig. S1A). A low-resolution structure of the CSU lattice was used for template matching in emClarity (17). The resulting convolution maps (Fig. S1B and S1C; Movie S2) display cross-correlation peaks for the selection of sub-tomograms (Fig. S1D and S1E). Although the template-matched subtomograms all contained six receptor trimers in a hexagonal lattice (Fig. S1Aiii and Fig. S1E), they were heterogeneous at the CheA/CheW baseplate level and resulted in four structural classes (Fig. S1Aiv). Sub-tomograms centered on a CSU trimer (Fig. S1Aiv, classes with green checks) were selected (Fig. S1F) and subjected to further refinement (Fig. S1A-v), from which sub-tomograms containing a single CSU were iteratively aligned and averaged to give rise to the final CSU map (Fig. S1A-vi).

**FIG 2:**
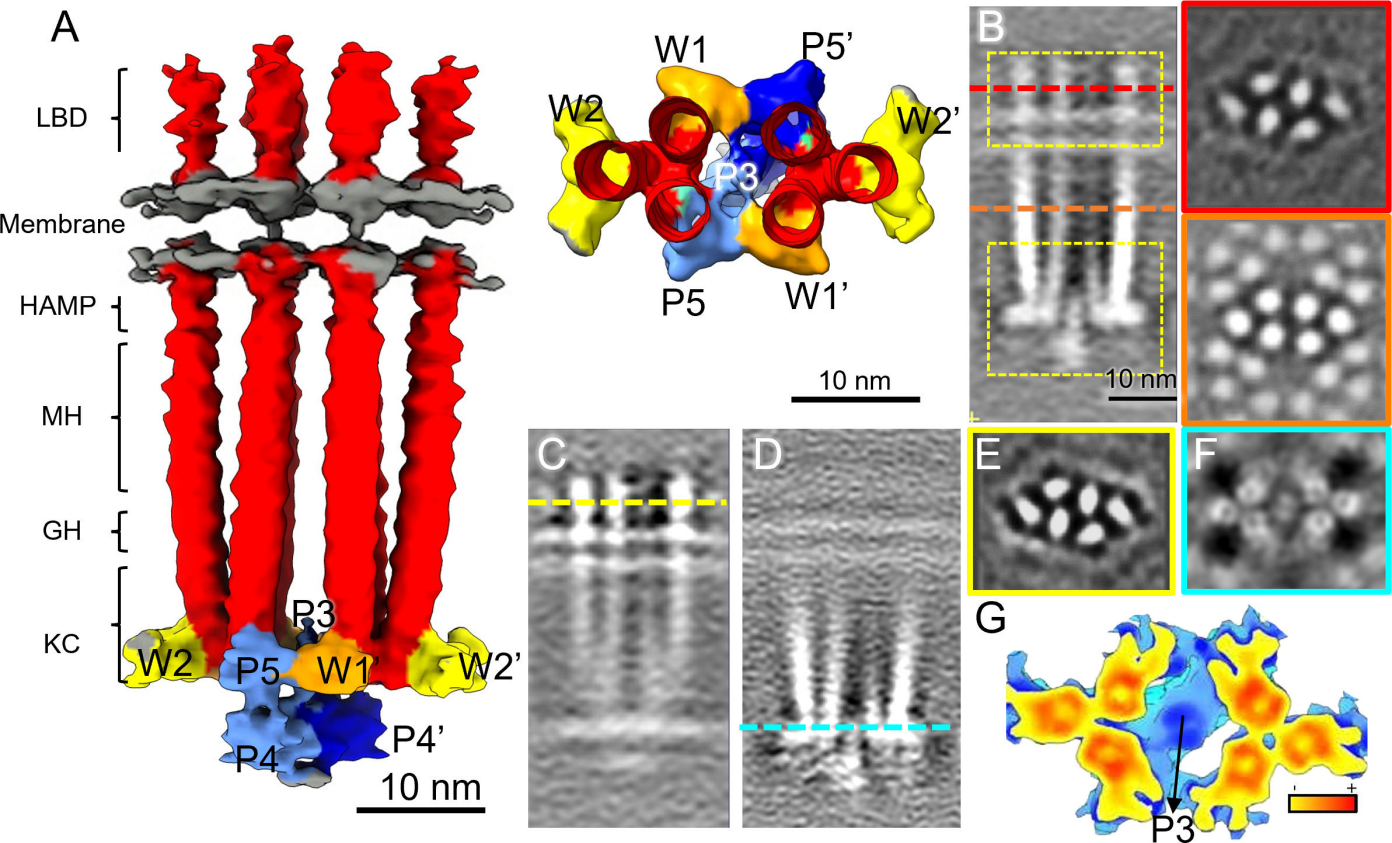
CryoET sub-tomogram averaging of the core signaling unit (CSU) of native chemosensory arrays. (**A**) Surface rendering of the CSU density map. Densities corresponding to the receptors are colored in red, CheA dimer in light/dark blue, CheW in yellow/orange, and lipid headgroups in gray. Specific regions of the receptors are labeled, including the ligand-binding domain (LBD), HAMP, methylation helix bundle (MH), glycine hinge (GH), and the kinase control region (KC). Individual CheA domains and CheW molecules are labeled accordingly. On the right side, a top view of the CSU from the glycine hinge downwards is displayed. (**B**) The CSU density map is shown in sectional side view (left) and top views (right). The top views are presented at the LBDs (red line) (top right) and at the cytoplasmic domains (orange line) (bottom right). (**C and D**) Focused alignment and averaging centered on the receptor LBDs (top dashed yellow box in panel B) (**C**) and on the baseplate region containing CheA, CheW, and the receptor signaling domain (bottom dashed yellow box in panel B) (**D**). (**E and F**) Sectional views of the averaged maps from C and D at the receptor LDBs (yellow line in panel C) (**E**) and baseplate (blue line in panel D) (**F**). Note the red and yellow lines in panels B and C, respectively, correspond to the same LBD position. (**G**) An enlarged view of the refined CSU baseplate, colored from blue to red, according to the density value.

The map of the full CSU, determined at an overall resolution of 12 Å (Fig. 2A; Fig. S2A), clearly resolves six full-length chemoreceptor dimers (Fig. 2A, red), a central CheA dimer containing distinct regions for P3–P5 (Fig. 2A, blue), four CheW monomers (Fig. 2A, orange and yellow), and the lipid bilayer headgroups (Fig. 2A, gray). The cytoplasmic organization of the complex is consistent with the recently reported CSU structure at 16-Å resolution from *E. coli* minicells (13) (Fig. S3A through S3C). Both maps show similar splaying of the receptors within each TOD as well as comparable baseplate densities, particularly regarding the shape and orientation of a CheA “keel” density that contains the P4 domain (Fig. S3C). The improved resolution of the present map, however, provides better localization of individual protein domains throughout the structure but particularly within the membrane-proximal region, especially the MCP LBDs (Fig. S3A and S3B). We note that while our biological samples contained wild-type receptors of all types, albeit carrying potentially variable methylation states, we are not able to distinguish different receptor types at the present resolution.

In the baseplate region, we further observed additional keel density apparently not attributable to CheA.P4. Comparison of the present CSU structure with the one derived from *in vitro* monolayer arrays at 8 Å (14) shows that the latter structure does not contain corresponding density in this region (Fig. S3D through S3G). Notably, the monolayer system utilized truncated CheA molecules not possessing P1 and P2 domains, suggesting that the excess density likely corresponds to these domains. Docking of the molecular model derived from the *in vitro* system (PDB 6S1K) (14) allowed further localization of the excess density, positioning it directly between and below the two P4 domains (Fig. S3H).

### Focused density refinement of the CSU periplasmic and baseplate regions

Local resolution analysis of the full CSU suggested that the complex was quite flexible in the periplasmic and CheA.P4 regions (Fig. S2B). We, therefore, carried out a focused refinement of the periplasmic and baseplate regions separately (Fig. 2B, yellow boxes). Indeed, as the density of each localized region improved during focused refinement, the remainder of the complex became poorly resolved (Fig. 2C and D), suggesting considerable structural flexibility between these two regions. The focused refinements also resolved additional structural details in both regions. In the periplasmic-focused map, the ellipsoidal shape of the individual LBDs became prominent and clearly indicated the orientation of each LBD (Fig. 2E), a feature not discernible in previous studies. These ellipsoidal densities showed a pseudo-threefold symmetry within each TOD, although no such symmetry was applied during refinement. Similarly, in the baseplate-focused map, the individual α-helices in the receptor signaling domain and CheA P3/P3′ dimerization domains became better resolved (Fig. 2F and G). Notably, the flexibility increases continuously from Gly hinge to methylation helices, and to the HAMP domain (Fig. 2D; Fig. S4A and S4B). Moreover, further refinement focused on the HAMP domain suggests flexible hinges on either side of the HAMP domain (Fig. S4C through S4E).

### All-atom model of the complete *E. coli* CSU

**FIG 3:**
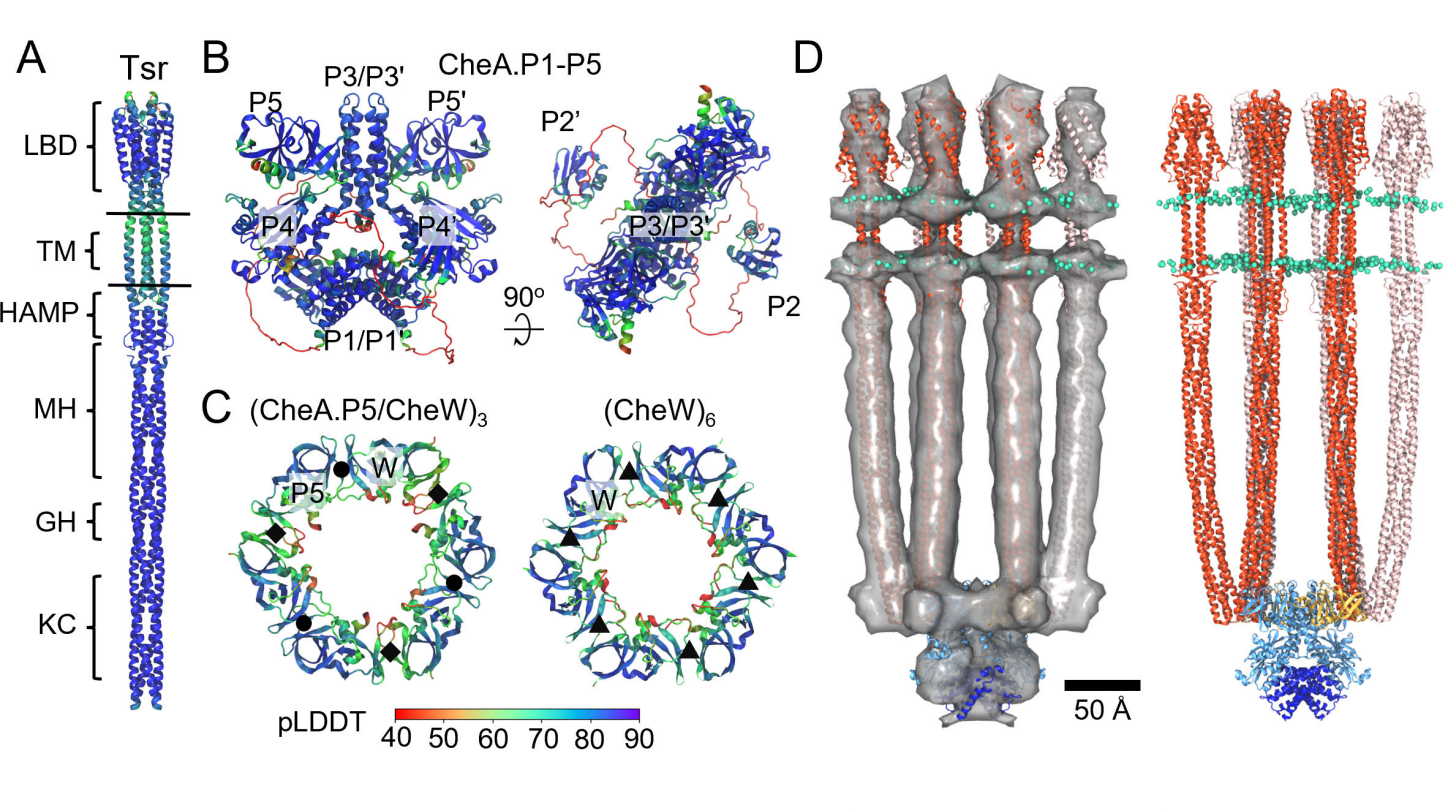
All-atom model of the full-length *E. coli* core signaling unit (CSU). (**A–C**) AlphaFold2 models of *E. coli* Tsr (**A**), CheA (**B**), and intact (CheA.P5/CheW)_3_ and (CheW)_6_ rings (**C**). Individual domains are labeled as in Fig. 2. Models are colored by pLDDT, a residue-based pLDDT confidence score. In panel C, interfaces I, II, and III are denoted by black circles, diamonds, and triangles, respectively. (**D**) Molecular dynamics flexible fitting-refined CSU model with (left) and without (right) overlayed density map. Tsr dimers are shown in red/pink; CheA.P3-5 is shown in light blue, CheA.P1 in dark blue, CheW in gold, and lipid headgroups in cyan.

Existing high-resolution information on the structures of full-length chemotaxis proteins is sparse. Previous *E. coli* structural studies have largely relied on the use of analogous structures from *Thermotoga maritima*, a distantly related organism, either directly or as templates for homology modeling, to assess residue-level details. Recently, AlphaFold2 (18) has been shown to produce high-fidelity atomistic structures of proteins and complexes using machine learning, providing a powerful alternative means of obtaining high-resolution structural information, especially in experimentally challenging cases. Accordingly, we used AlphaFold to construct models of the *E. coli* CSU proteins and sub-complexes, including full-length Tsr and CheA as well as the (CheA.P5/CheW)_3_ and (CheW)_6_ hexameric rings comprising the array baseplate (Fig. 3A through C). In all cases, wild-type sequences were used. Overall, the predicted structures are of high quality as assessed by pLDDT, a per-residue confidence score output by AlphaFold, and agree well with existing high-resolution structures and previous modeling results as described below.

The predicted full-length Tsr structure (Fig. 3A) is consistent with X-ray crystal structures of the Tsr cytoplasmic domain (PDB 3Z X6) (19) and APO Tsr periplasmic domain (PDB 2D4U) (20). Additionally, the Tsr TM, control cable, and HAMP regions, which were previously unresolved, closely resemble those seen in a crystal structure of the symmetric APO state of the NarQ sensor kinase (PDB 5JEQ) (21) and agree well with previous modeling results based on TM cross-linking data from *E. coli* Tar (13, 22). In particular, the predicted TM bundle displays a central TM1/TM1′ dimer with flanking TM2 helices, as previously proposed for *E. coli* chemoreceptors generally (23), and the critical control-cable segment connecting TM2 to the AS1 helix of HAMP is observed to be helical and kinked as previously suggested based on mutagenesis data (24).

The predicted folds of the individual *E. coli* CheA domains and their inter-domain arrangements (Fig. 3B) agree well with existing crystal structures from *T. maritima* (25, 26). The P3 and P4 domains adopt a planar arrangement similar to that seen in a crystal structure of a soluble *T. maritima* CheA.P3-P4 construct (PDB 4XIV) (26) and closely resemble the ‘undipped’ CheA conformation deduced from cryoET maps of *in vitro* monolayer arrays (PDB 3JA6 and 6S1K) (7, 14). The P5 regulatory domains are rotated slightly compared to the orientation required to form interface I with CheW. Intriguingly, the P1 domains were observed to form a dimer wedged between the two P4 domains (Fig. 3B) where the aforementioned excess density in our cryoET map is localized (Fig. 3D). The locations of the P2 domains, on the other hand, vary considerably between AlphaFold predictions (Fig. S5). The functional implications of these observations are discussed in more detail below. Additionally, in line with the disordered nature of the long, flexible linkers connecting P1 and P2 to one another and P3 (27), AlphaFold predicts these regions to be unstructured with low pLDDT score (Fig. S6).

In the case of the (CheA.P5/CheW)_3_ ring (Fig. 3C), which contains interfaces I and II between CheA.P5 and CheW, the AlphaFold-predicted structure agrees well with the analogous *T. maritima* crystal structure (PDB 4JPB) (28), as well as *in vivo* cross-linking studies in *E. coli* (29). Additionally, the predicted (CheW)_6_ ring structure is in good agreement with molecular models derived via analogy with the (CheA.P5/CheW)_3_ ring and validated with *in vivo* cross-linking data (8, 30).

To aid in the computational assembly of the CSU, we also utilized AlphaFold to construct models of the other key protein-protein interfaces, including the trimeric interface between the Tsr protein-interaction regions as well as the Tsr/CheA.P5 and Tsr/CheW interfaces. The constituent models were assembled via rigid docking using the predicted interfaces and our cryoET map to produce a preliminary model of the CSU, which was subsequently refined using molecular dynamics flexible fitting (MDFF) (31) (Fig. S7). Finally, the refined CSU model was embedded in an atomistic lipid bilayer (Fig. 3D) with a composition chosen to mimic that of the *E. coli* inner membrane (32). Full details regarding the modeling procedures are provided in the Materials and Methods.

### Neighboring receptors form transient interactions within the periplasmic space

**FIG 4:**
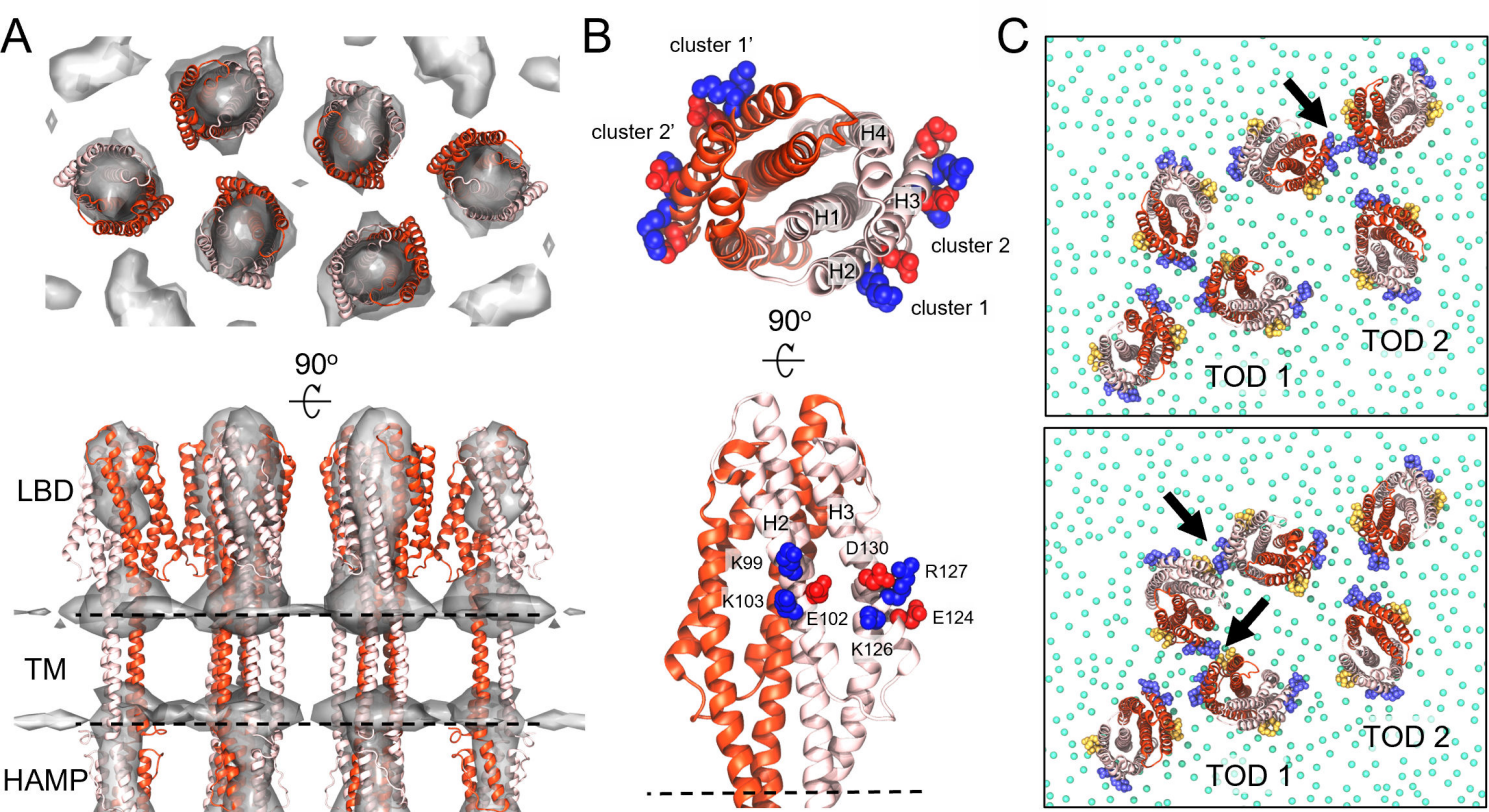
Periplasmic inter-receptor interactions within CSU. (**A**) Top and side views of the periplasmic region of the refined CSU model, involving the receptor ligand-binding domain (LBD), transmembrane (TM) bundle, and HAMP domain, overlayed with the cryoET STA map (shown in Fig. 2C and E). Receptor monomers are differentiated using light and dark red. The location of the membrane, not shown for clarity, is illustrated with dashed lines. (**B**) Top and side views of a single Tsr LBD, highlighting the two clusters of residues observed in an MD simulation to form inter-receptor interactions. Individual residues within each cluster are labeled and shown as Van der Waals (VDW) spheres in red (acidic) or blue (basic). LBD helices are labeled H1–H4. (**C**) Snapshots from an MD trajectory illustrating interactions (arrows) between receptors within the same trimer of dimers (TOD) (top) and between TODs (bottom). For clarity, residues forming clusters 1 and 2 have been colored yellow and purple, respectively, and only the headgroup of each lipid is shown (green spheres).

The improved characterization of MCP LBD orientations in our cryoET STA map permitted positioning these domains within the full CSU structure with added confidence (Fig. 4A). To test the robustness of the obtained fits, we conducted a series of MDFF simulations in which the receptor LBDs were rotated by 30°, 60°, or 90° from their initial positions. Indeed, in each simulation, the LBDs returned to their original fitted orientations. We additionally noted potential interactions between neighboring LBDs as they rotated past one another. We therefore decided to carry out an all-atom simulation of the CSU to explore the possibility of specific inter-receptor interactions within the periplasmic space. The resulting trajectory (Movie S3) identified interactions between two co-planar clusters of charged residues on the Tsr LBDs, involving K99, E102, and K103 on helix 2 (cluster 1) as well as E124, K126, R127, and D130 on helix 3 (cluster 2) (Fig. 4B). In addition to interactions between receptors within the same TOD, we also observed interactions between the two TODs (Fig. 4C). Given the symmetry of the extended array architecture, especially in the periplasmic space (30), such interactions presumably also occur between receptors from neighboring CSUs. Notably, however, the observed interactions were transient (<10 ns) and did not form a single, long-range pattern across the CSU on the timescale of our simulation. Rather, these interactions appear to form a pseudo-lattice by which the Tsr LBDs roughly maintain their relative orientations and inter-domain distances.

### CheA.P1 forms an anti-parallel dimer that interacts non-productively with CheA.P4

**FIG 5:**
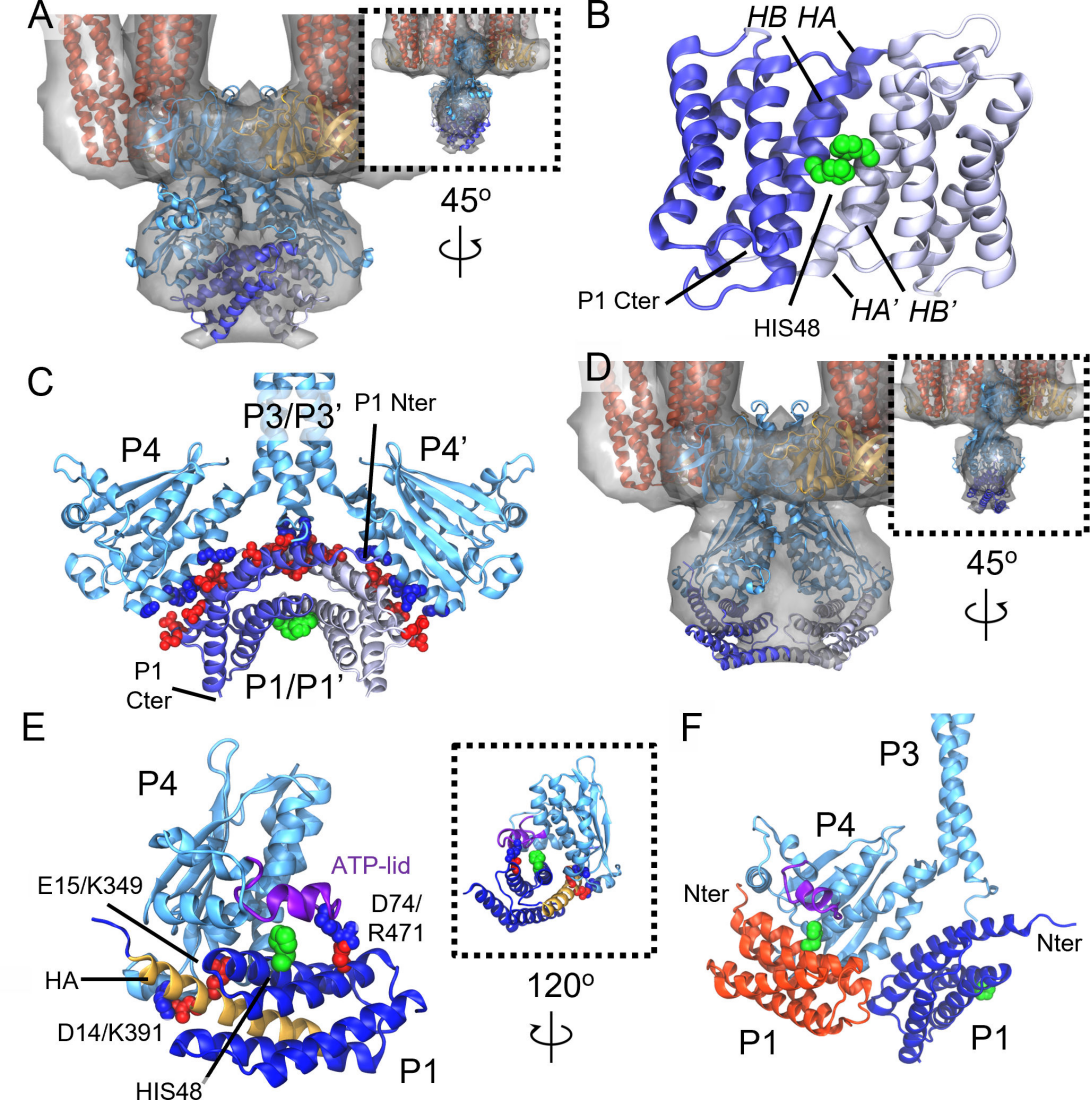
CheA.P1/P1 and CheA.P1/P4 interactions within the CSU. (**A**) Baseplate region of the MDFF-refined CSU model with density map overlayed, illustrating the fit of the AF2-predicted P1 dimer bound non-productively to P4/P4′. (**B**) Anti-parallel P1 dimer structure predicted by AF2 for full-length CheA shown in isolation. The individual P1 monomers are depicted in light and dark blue color and the substrate histidines are colored green and shown in VDW representation. The A and B helices mediating the dimer interaction are labeled for each monomer. (**C**) Predicted interaction interface between P1 and P3-P4. Acidic and basic residues forming potential inter-domain salt bridges are shown in red and blue, respectively. Note: the surrounding regions of the CSU model have been removed for clarity. (**D**) Baseplate region of the CSU in which a model of the dipped CheA conformation bound productively to P1 has been docked into the overlayed density map. (**E**) Predicted interaction between CheA.P1 and CheA.P4 for the productive transfer of phosphate from bound ATP (not shown) to HIS48 (green VDW spheres). Residues stabilizing the interaction are labeled and shown as VDW spheres in red (acidic) or blue (basic). CheA.P1 and CheA.P4 are shown in dark and light blue, respectively. P1 helix A and the ATP-lid are shown in gold and purple, respectively. (**F**) Overlay of non-productive (blue) and productive (red) P1/P4 binding modes. The N-terminus of each is labeled.

The central P1 dimer predicted as part of the full-length CheA structure by AlphaFold describes well the excess density between the P4 domains observed in our map (Fig. 5A), which was previously noted within a cryoET map from *E. coli* minicells but could not be assigned atomic structure (13). Moreover, the size and shape of the P1 dimer suggest it alone accounts for this excess, in line with the variable placement of the P2 domains by AlphaFold. The apparent lack of P2 density therefore suggests that it may not form stable, functional interactions with the rest of the complex, an observation in line with both solution NMR measurements (33) as well as studies involving P2-deletion CheA mutants demonstrating that P2 is not strictly required for effective kinase regulation and chemotaxis (34). The P1 domains themselves are predicted to interact in an anti-parallel fashion mediated by P1 helices A and B, such that the substrate histidines face away from the P4 catalytic site and toward the P1/P1′ interface (Fig. 5B). The interaction between P1 and P4 is primarily mediated by a string of oppositely charged residues residing on helix D and the C-terminus of helix A in P1 and the α3 helix in P4 (Fig. 5C). Additionally, there exist interactions between the N-terminus of P1 helix A and the N-terminus of the P3 dimerization bundle, particularly between the previously noted P1 residues D14 and E18 (35) and P3 residue R265, which has been shown to play a vital role in CheA function (7, 36). Such interactions may also account for the observed increase in *E. coli* CheA dimer affinity attributed to the presence of P1 (37).

In line with this predicted P1/P4 binding surface, a previous NMR analysis of *T. maritima* CheA, designed to monitor the binding of free P1 to a soluble P3/P4 construct, showed large chemical shifts on the P1 D helix as well as on the P4 α4 helix, a region distal to the catalytic site, leading to the proposal of a non-productive P1/P4 binding mode (38). In addition, sizable changes in chemical shifts were also reported on the P1 A and B helices, which form interactions with both P4 and the neighboring P1 monomer in our predicted structure, while the P1 C and E helices, which showed little change in chemical shifts, do not form any inter-domain contacts. In contrast, the predicted CheA structure primarily shows the P4 α3 helix mediating non-productive P1 interactions, whereas the NMR analysis suggested the P4 α4 helix fulfills this role. It is possible, however, that this discrepancy may arise due to slight alterations in the relative orientations of the P3 and P4 domains due to the soluble and truncated nature of the CheA constructs used in the NMR analysis.

Recently, Muok and co-workers also observed close interactions between the P1 domains within *T. maritima* ‘foldon’ complexes, especially in the inhibited state, and further reported a crystal structure of a *T. maritima* P1 dimer (39). Like the AlphaFold-predicted *E. coli* P1 dimer in full-length CheA, this structure showed the substrate histidines facing toward the dimer interface in a non-productive fashion; however, the P1 domains adopted a parallel configuration as opposed to an anti-parallel one. Indeed, for two isolated *E. coli* P1 monomers, AlphaFold predicts a parallel arrangement similar to that reported in *T. maritima* (Fig. S8), although the relatively low Predicted Aligned Error (PAE) scores (Fig. S8) between P1 monomers suggests that AlphaFold is not confident in this predicted arrangement. We note, however, that this does not necessarily suggest the interface is incorrect but rather that it should not be interpreted on its own.

In light of the above observations, we wondered how well a parallel P1 dimer configuration would agree with the density map. We therefore rigidly docked the parallel P1 dimer predicted by AlphaFold into the excess density between the P4 domains (Fig. S9). Despite the excess density being relatively featureless, the parallel P1 dimer fit is considerably poorer than the anti-parallel configuration in terms of overlap with the density. Additionally, there do not appear to be any potential strong contacts to be formed between P1 and P4. Nevertheless, we cannot rule out that changes in the conformations of P4 and/or at the P1 dimer interface might produce alternate dimer configurations that describe well the density. Along these lines, analysis of the PAE plot for full-length CheA (Fig. S6) suggests that AlphaFold is relatively confident in its prediction for the inter-domain arrangement between P1 and P4 but less confident in the arrangement between the two P1 domains. We suggest therefore that important, non-productive interactions between P4 and P1 drive the dimerization of P1 in the spatially constrained environment of the kinase core. Whether P1 also dimerizes away from the kinase core and the particular configuration such a dimer might acquire will require future studies to elucidate.

### Keel density can accommodate dipped CheA.P4 with productively bound P1

Previously, we predicted the existence of a “dipped” P4 conformation using molecular simulations of the *T. maritima* CSU (7), which we also subsequently identified in a sub-nanometer cryoET map of the *E. coli* CSU from *in vitro* monolayer arrays (14). Recently, multiple experimental studies have lent strong evidence to the existence of such a conformation and demonstrated its importance for the CheA catalytic cycle (5, 36, 40). Although the present resolution prevents explicitly isolating distinct P4 conformations within our cryoET map, a rigid docking of our dipped CheA model is also consistent with the density (Fig. 5D). However, the dipped P4 domains, which are rotated downward and toward one another, considerably reduce the volume attributable to the P1 dimer and prevent the formation of predicted stabilizing interactions with P4, suggesting that the postulated non-productive binding of a P1 dimer is not compatible with a dipped P4 conformation. We therefore explored what implications this conformational change might have on the productive binding of P1 to P4.

Previous molecular docking (41) and mutagenesis (35) studies have led to models for the productive interaction of P1 with P4, which involve primarily P1 helices A and B oriented in such a way as to permit the highly constrained spatial requirements for chemical transfer of the gamma-phosphoryl group from bound ATP to the substrate histidine on P1 helix B (42). Additionally, a previous crystal structure of *T. maritima* CheA.P4 bound to an ATP analog showed that the so-called ATP lid, a typically unstructured span of ~20 residues near the nucleotide binding site, adopted an ordered conformation, thereby altering the probable interface for productive P1 binding (43). This observation led to the proposal that folding of the ATP lid is a prerequisite for productive P1 binding, a notion which has been further supported by recent kinetic analyses of CheA activation, suggesting an ordered sequential mechanism (44).

To assess the implications of the above observations, we constructed a model of the productive P1/P4 binding mode for *E. coli* CheA. As AlphaFold is currently not able to predict coordinates for ligands and co-factors when folding protein structures, it does not capture the folded ATP lid conformation induced by ATP binding to CheA.P4. We therefore opted to construct a homology model of *E. coli* P4 with a folded ATP lid based on PDB 1I58 and use HADDOCK (45) to carry out an extensive exploration of potential P1/P4 interactions. During the docking procedure, the distance between the substrate histidine and gamma-phosphoryl group was restrained to between 2 and 5 Å to encourage productive binding orientations. The top-ranked prediction (Fig. 5E) visually agrees with the productive P1/P4 binding mode proposed for *T. maritima* CheA (41) and shows interactions mediated primarily by P1 helices A and B, including salt bridges between residues D14/K391 and E15/K349, as well as a potential salt bridge between D74 on P1 helix C and R471 on the P4 ATP lid. Thus, P1 helix A appears to play an important role in both productive and non-productive P1 binding (Fig. 5F), although the N-terminus faces inward toward the P3 bundle in the non-productive binding mode and is outward facing in the productive binding mode. Notably, the proximity of the productive and non-productive P1 binding modes (Fig. 5F) further suggests there may be some degree of steric hindrance to simultaneous binding, an observation that may be relevant to studies involving the use of liberated P1 domains to assess CheA signaling mechanism (44, 46, 47).

Finally, we used our model of the productive P1/P4 binding mode to map P1 onto the dipped CheA structure, producing an overall CheA configuration that is fully accommodated by the keel density (Fig. 5D). Unlike the non-productive binding of P1 to undipped P4, however, which poises the P1 domains to bury a sizable surface area through dimerization, the productively bound P1 domains do not. Rather they face one another through a small cross-sectional area formed by the turns connecting helices A with B and C with D, suggesting that P1 dimerization likely does not play an important role in the productive P1/P4 interaction. However, as we have only modeled a single dipped P4 conformation here and have assumed an overall symmetric CheA configuration for simplicity, future work will be required to characterize potential P1/P1 interactions when productively bound to P4.

## DISCUSSION

In this study, we have reported the structure of the chemotaxis CSU from E protein lysed *E. coli* cells at an overall resolution of 12 Å by cryoET STA. Using AlphaFold2, we have further predicted the full-length structures of the CSU’s constituent proteins as well as key protein-protein interfaces, enabling the assembly an atomistic CSU model, which we conformationally refine using our cryoET data. The improved resolution in the periplasmic region allowed for a more precise localization of the positions and relative orientations of the receptor LBDs. An MD simulation of the CSU model in an atomistic lipid bilayer permitted us to directly observe the local diffusion of receptors within the membrane for the first time and identified clusters of charged residues forming interactions between neighboring receptor LBDs. In line with these observations, previous *in vivo* work using a Tar-only *E. coli* strain observed that the efficiency of disulfide cross-linking between residues in periplasmic helices 2 and 3 varied as a function of methylation state and exposure to the chemoattractant aspartate (48). However, analysis of the cross-linking results was based on a crude picture of CSU structure and array architecture, potentially limiting functional interpretations. Our present model and observations therefore provide a high-resolution structural basis for designing experiments to test for concerted, signal-dependent changes in the periplasmic space.

Additionally, integration of AlphaFold2 predictions of full-length CheA into our cryoET density map allowed us to incorporate the P1 domains into the CSU model as an anti-parallel dimer, non-productively bound between the P4 domains. As previously noted, this prediction is in line with findings in *T. maritima*, which suggest both the existence of a non-productive P1-P4 binding mode and P1 dimerization (38, 39). Moreover, previous *in vivo* cryoET images of *E. coli* CSUs in distinct signaling states imposed by mutagenesis showed that the overall volume of CheA density tended to increase in the kinase-OFF state, suggesting that regulation of P1 and P2 mobility might be a key feature of kinase control (49). Along these lines, the non-productive P1/P4 contact interfaces presented here require both P4 domains to adopt an undipped conformation, which we previously showed to be stabilized by direct P4-P5 contacts (14). The P1 dimer may therefore act as a wedge to further reduce P4 mobility and CheA dynamics overall in the kinase-OFF state. Our results suggest, however, that P2 is not integral to this ordered state, and thus, its mobility is unlikely to be directly regulated.

Studies involving the kinetic analysis of CheA autophosphorylation using liberated P1 domains, on the other hand, have discounted the role of P1 sequestration in CheA regulation, proposing that receptors primarily mediate kinase regulation by altering the apparent rate constant of autophosphorylation through control of ATP binding (44, 47, 50). Nevertheless, our present observations can be reconciled with such a picture if, for instance, ATP binds preferentially to either the undipped or dipped P4 conformation, thereby allowing receptors to indirectly mediate ATP binding through the regulation of P4 dynamics, which could be in turn affected by P1 dimerization and sequestration. It has been previously noted that the undipped P4 conformation situates the unfolded ATP-lid near the P5 regulatory domain where it can form stabilizing interactions (5, 14). One possibility, therefore, is that such interactions discourage the folding ATP-lid, preventing stabilization of ATP binding and the formation of a suitable interface for productive P1 binding (41, 43). Another possibility is that differences in the P3-P4 and P4-P5 linker conformations, which have previously been shown to be critical for kinase function and coupled to P4 dipping (40, 51 – 53), might induce subtle changes in the ATP-binding pocket that increase ATP binding affinity for the dipped P4 state. Given that P4 dipping would also give rise to an overall CheA configuration that destabilizes the binding of a non-productive P1 dimer, such a conformational change should ultimately lead to disassociation of the P1 dimer, freeing it for productive binding to P4.

Moving forward, the use of strategic functional mutations combined with the improved resolutions enabled by cryoET analysis of E-lysis and minicell constructs will provide a promising tool to reveal critical details of signaling mechanisms such as kinase regulation. The overall similarity between present CSU map and that obtained from *E. coli* minicells (13) suggests that insights from both contexts may be transferrable. Additionally, our atomistic CSU model will greatly enhance the potential for the investigation of signaling mechanisms using molecular simulation, providing a unique way to obtain high-resolution mechanistic insight.

## MATERIALS AND METHODS

### Bacterial strains, plasmids, cell culture, and cryoEM sample preparation

The pRY100 plasmid, carrying the phage φX174 lysis E gene under a tacP promoter and a lacIQ repressor control (54), was transformed into the wild-type *E. coli* K-12 strain RP437 cells using a standard protocol (55). For fluorescence imaging, cells were also transduced with a CheY-GFP construct. Cells were grown in the TB broth (1% tryptone and 0.4 NaCl, pH 7.0) supplemented with 100-µg/mL ampicillin. E gene expression was induced by addition of 0.5-mM isopropyl β-D-thiogalactopyranoside at an OD_600_ of 0.6 as previously described (15). Ten minutes after induction, an aliquot of 4-µL culture, mixed with 1-µL fiducial gold beads (10-nm size), was applied to glow-discharged Quantifoil *R*2/2 grids and vitrified with a GP2 plunge-freezing device (Leica).

### Cryo-ET data acquisition

The cryoET titled series data were collected using a Thermo Fisher Titan Krios G3 instrument, with a K2 summit detector at a pixel size of 2.8 Å and a K3 summit detector at a pixel size of 2.1 Å (see Table S1). Data were collected using the SerialEM software (56, 57).

### Cryo-ET reconstruction and sub-tomogram averaging

Motion correction was performed using MotionCor2 (58). The data collected by the K3 detector were rescaled to 2.8 Å/pixel by Fourier cropping in MotionCor2. The tilt series were aligned using fiducial gold beads in Etomo (59). The aligned tilt series were then imported into emClarity (17) for contrast transfer function (CTF) correction and weighted backprojection (WBP) reconstruction. About 15% of the analyzed tomograms contained arrays of CSUs. A total of 33 array-containing tomograms were selected for cryoET sub-tomogram averaging.

Template matching as implemented in emClarity was used to for sub-tomogram particle picking. The position and orientation of picked particles were plotted for visual inspection, and bad particles were manually removed. The resulting sub-tomograms were then aligned and classified according to the workflow described in Fig. S1. A total of 950 CSU trimers from the K2 data set processed using emClarity initially yielded an average structure at 8.8-Å resolution but with a strong preferred orientation. A second data set was collected with a K3 detector, and additional sub-tomograms containing side views were added. In addition, single CSU sub-volumes were extracted from the CSU trimers through symmetry expansion and were subjected to further alignment and refinement using i3 (60).

### Molecular modeling and simulations

Atomic models of the CSU’s constituent proteins and interfaces were predicted using AlphaFold-Multimer v.3 (18, 61) via the ColabFold (62) *AlphaFold2_mmseqs2* notebook at https://github.com/sokrypton/ColabFold. Structural templates were not explicitly used for any of our AlphaFold predictions. A preliminary CSU model was assembled via rigid docking of the component models using UCSF ChimeraX (63). Breifly, this process was carried out by first constructing a model of the Tsr TOD using AlphaFold models of a full-length Tsr homodimer and trimer of Tsr protein-interaction regions. As the receptor C-termini (residues 517–551) were predicted to be unstructured by AlphaFold and accordingly did not have corresponding density within the cryoET map, we excluded them from further modeling. Two copies of the Tsr TOD model were then rigidly docked into our cryoET density map followed by two copies each of the (CheA.P5/CheW)_3_ and (CheW)_6_ hexameric ring models. Finally, a model of the CheA homodimer, excluding the CheA.P2 domain due to its variable placement within the AlphaFold models, was docked. Manual rearragements were required only for the CheA.P5 domain, whose positioning in the homodimer model was not consistent with interface 1. We therefore removed the P5 domains from the homodimer model and reattached the P4-P5 linker to the P5 domains present within the (CheA.P5/CheW)_3_ ring models. The resulting Tsr/CheA.P5 and Tsr/CheW interactions were checked using AlphaFold models of the respective interfaces. Finally, the entire structure (Fig. S5) was conformationally refined to our cryoET data through a series of symmetry-restrained MDFF simulations (31). All MDFF simulations were carried out in NAMD v.2.14 (64, 65) and performed in the NVT ensemble at 300 K with a coupling factor of 0.3 applied to the protein backbone. Additional harmonic restraints were applied during fitting to prevent the loss of secondary structure as well as the formation of cis-peptide bonds and chirality errors. The stereochemistry of the entire CSU model was then refined using ISOLDE (66) in ChimeraX and validated using MolProbity (67).

To prepare the refined CSU model for MD simulation, an atomistic lipid bilayer consisting of 70% PVPE (1-palmitoyl 2-cis-vaccenic phosphatidylethanolamine), 20% PVPG (1-palmitoyl 2-cis-vaccenic phosphatidylglycerol), and 10% cardiolipin (1-palmitoyl 2-cis-vaccenic 3-palmitoyl 4-cis-vaccenic diphosphatidylglycerol) was assembled around the transmembrane region using CHARMM-GUI (68). The resulting structure was then solvated with TIP3P (69) water molecules and 150-mM KCl and subjected to conjugant gradient energy minimization followed by a series of equilibration simulations conducted as follows. First, solvent and lipids were permitted to relax, while the full protein was harmonically restrained following the multi-step equilibration protocol recommended by CHARMM-GUI. Next, restraints on the protein structure were slowly removed over a span of 10 ns by lowering the associated spring constants in increments of 2 ns. Finally, a 120-ns production simulation without restraints was carried out with analyses being conducted on the last 100 ns. All MD simulations were performed using NAMD v.2.14 (64, 65) and the CHARMM36 force field (70). Production simulations were conducted in the NPT ensemble with conditions maintained at 1 atm and 310 K using the Nosé-Hoover Langevin piston and Langevin thermostat, respectively. The r-RESPA integrator scheme was employed with an integration time step of 2 fs, and SHAKE constraints were applied to all hydrogen atoms. Short-range, non-bonded interactions were calculated every 2 fs with a cut-off of 12 Å; long-range electrostatics were evaluated every 6 fs using the particle-mesh Ewald method.

## Data Availability

All data needed to evaluate the conclusions in the paper are present in the paper and/or the supplemental materials. The cryoET STA map of the full CSU has been deposited in the EMDB under accession code EMD-15641. The cryoET STA maps of the CSU periplasmic and baseplate regions after focused refinement and classification have been deposited in the EMDB under accession code EMD-15643 and EMD-15642, respectively. Atomic coordinates for the associated CSU model are deposited in the PDB under accession code 8C5V.

## SUPPLEMENTAL MATERIAL

Supplemental material mbio.00793-23-s0001.pdf Supplemental figures, table, text, and movie captions.

Movie S1 mbio.00793-23-s0002.mp4 Aligned tilt series of an E protein lysed native *E. coli* cell displaying a patch of the chemosensory array lattice.

Movie S2 mbio.00793-23-s0003.mp4 A convolution map resulted from template-matching in emClarity, overlapped with picked subtomograms (green circles).

Movie S3 mbio.00793-23-s0004.mp4 Extract from MD simulation (50-ns) of CSU model in an atomistic lipid bilayer, illustrating interactions between neighbouring receptor ligand binding domains (LBD). Clusters of residues mediating LBD interactions are shown as VDW spheres and colored in blue (residues E124, K126, R127, D130) or yellow (residues K99, E102, and K103).

ASM does not own the copyrights to Supplemental Material that may be linked to, or accessed through, an article. The authors have granted ASM a non-exclusive, world-wide license to publish the Supplemental Material files. Please contact the corresponding author directly for reuse.
