# Supplementary material for: Structure of the native chemotaxis core signaling unit from phage E-protein lysed E. coli cells
Source: mBio. 2023 Sep 29;14(5):e00793-23. doi: 10.1128/mbio.00793-23 (PMC10653900; doi:10.1128/mbio.00793-23)
Supplement: Supplemental material — Supplemental figures, table, text, and movie captions. [file mbio.00793-23-s0001.pdf]

Supplementary materials

for

Structure of the Native Chemotaxis Core Signalling Unit from E-gene lysed *E. coli* cells

C. Keith Cassidy, Zhuan Qin, Thomas Frosio, Khoosheh Gosink, Zhengyi Yang, Mark Sansom, Phillip J. Stansfeld, John S Parkinson, Peijun Zhang

List of Supplementary materials

Supplementary Table 1
Supplementary Figure 1 - 9
Supplementary Movie 1-3

Supplementary Table 1 | CryoET data collection and structure determination of CSU from native *E. coli* cells.

<table>
<tr><td colspan="4">Data acquisition</td></tr>
<tr><td>Dataset</td><td colspan="2">Titan Krio (K2)</td><td colspan="2">Titan Krio (K3)</td></tr>
<tr><td>Voltage (keV)</td><td colspan="2">300</td><td colspan="2">300</td></tr>
<tr><td>Detector</td><td colspan="2">Gatan Quantum K2
Direct Electron Detector</td><td colspan="2">Gatan Quantum K3
Direct Electron Detector</td></tr>
<tr><td>Energy-filter</td><td colspan="2">Yes</td><td colspan="2">yes</td></tr>
<tr><td>Slit width (eV)</td><td colspan="2">20</td><td colspan="2">20</td></tr>
<tr><td>Super-resolution mode</td><td colspan="2">no</td><td colspan="2">yes</td></tr>
<tr><td>Pixel size (Å/pixel)</td><td colspan="2">2.8</td><td colspan="2">2.1</td></tr>
<tr><td>Defocus range (µm)</td><td colspan="2">-2 to -3</td><td colspan="2">-0.5 to -3</td></tr>
<tr><td>Acquisition scheme</td><td colspan="2">Dose-Symmetric, -60/60, 3° step, group 3</td><td colspan="2">Dose-Symmetric, -60/60, 3° step, group 3</td></tr>
<tr><td>Total Dose (electrons/Å^2)</td><td colspan="2">120</td><td colspan="2">120</td></tr>
<tr><td>Number of Frames</td><td colspan="2">8</td><td colspan="2">10</td></tr>
<tr><td>Number of Tomograms</td><td colspan="2">21</td><td colspan="2">12</td></tr>
<tr><td colspan="5">Structure determination</td></tr>
<tr><td>No. of subotomograms</td><td colspan="2">3596</td><td colspan="2">1509</td></tr>
<tr><td rowspan="2">Resolution (Å)</td><td>Full CSU</td><td colspan="2">periplasmic</td><td>baseplate</td></tr>
<tr><td>12.0</td><td colspan="2">12.5</td><td>11.0</td></tr>
<tr><td>Data deposited</td><td>EMD-15641
PDB-8C5V</td><td colspan="2">EMD-15643</td><td>EMD-15642</td></tr>
</table>

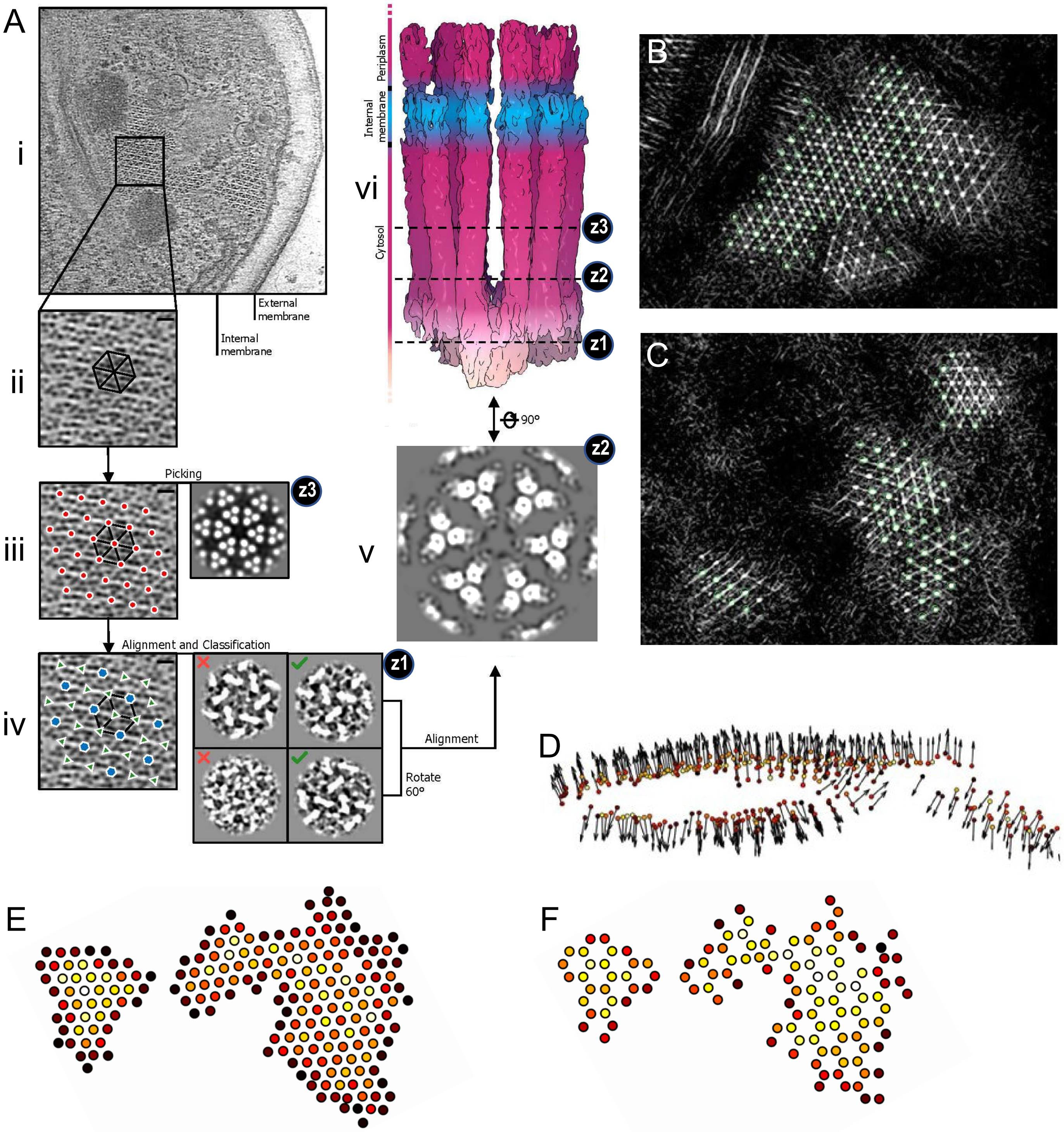

Supplementary Figure 1 | CryoET STA workflow for the CSU trimer. (A) Overview of the cryoET STA workflow. Z1 and Z2 indicate the positions of cross-sections. Steps are indicated as i) raw tomogram, ii) template matching, iii) subvolume picking (the average is shown on the right), iv) alignment and classification (four classes are shown on the right), v) average of CSU trimer, and vi) symmetry expansion and final average of a single CSU. (B-C) Two representative sections of the same conversion map overlapped with picked subvolumes (green circles). (D) Orientations of the picked subvolumes plotted with arrows. (E) Positions of subvolumes after picking in A (iii), where receptor signal dictates. (F) Positions of subvolumes after classification in A (iv), where CSU trimer classes are selected (green checks). The colors indicate cross correlation values, from lowest (black) to highest (white).

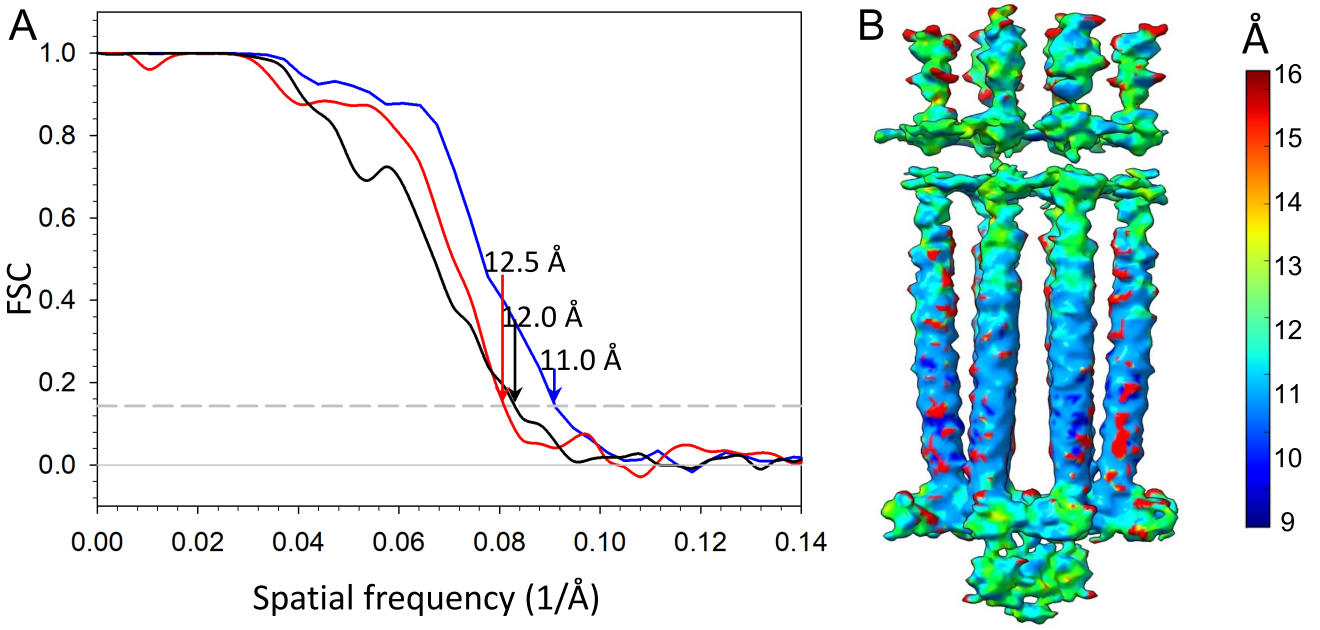

Supplementary Figure 2 | Fourier Shell Correlation (FSC) of the CSU subtomogram average. (A) FSC plots of the overall CSU (black), chemoreceptor ligand binding domains (red), and baseplate region (blue). (B) Local resolution map of the overall CSU, colored from blue to red as indicated in the key.

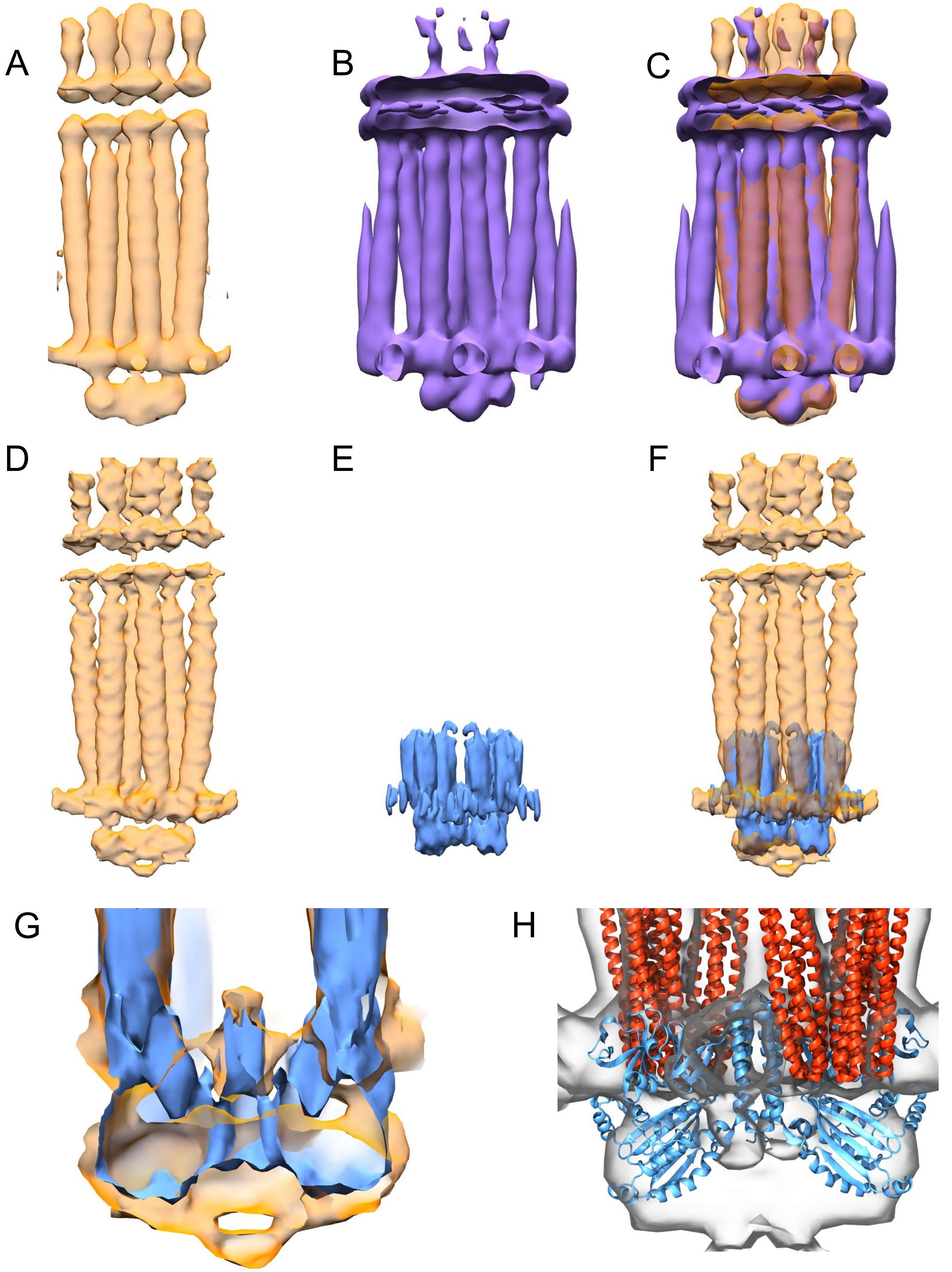

Supplementary Figure 3 | Comparison of CSU maps. (A) CSU map from *E. coli* ghost cells, low-pass filtered to 16 Å resolution. (B) CSU map at 16 Å resolution from *E. coli* minicells (EMD-10160). (C) Overlay of A and B. (D) CSU map from *E. coli* ghost cells, median filtered at 12 Å resolution. (E) CSU map from *E. coli* 4Q monolayer arrays (EMD-10050), low-pass filtered to 12 Å resolution. Note the P1 and P2 domains of CheA are not present in the monolayer sample. (F) Overlay of the D and E. (G) A slice through a zoomed view of (F), highlighting excess density between CheA P4 domains. (H) Overlay of CSU map from *E. coli* ghost cells with model constructed from 4Q monolayer arrays (PDB 6S1K). For clarity, CheW is not shown.

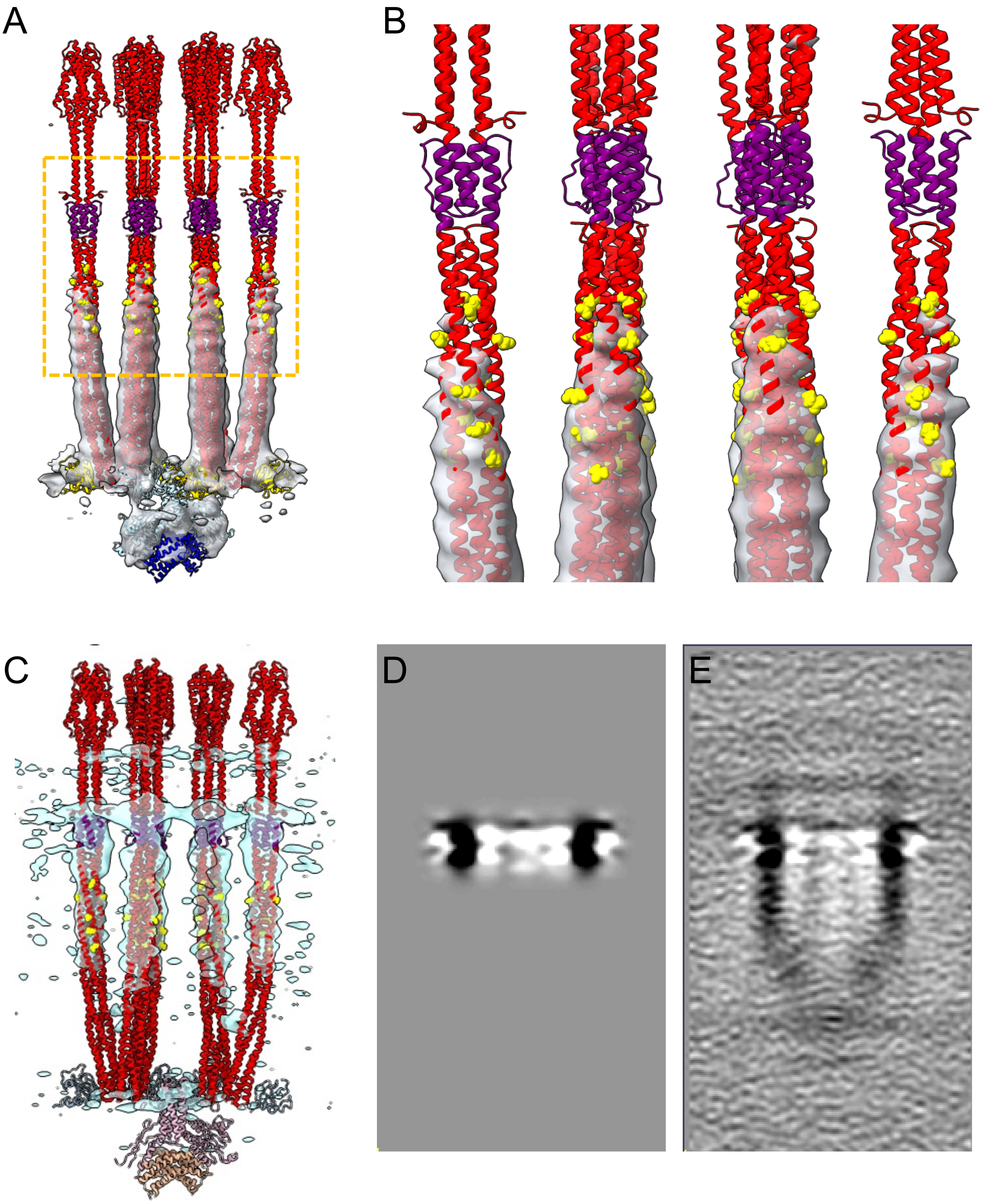

Supplementary Figure 4 | Baseplate- and HAMP-focused refinements. (A) Overlay between the CSU model and the baseplate-focused refined map. (B) Zoom onto region outlined by the dashed orange box in (A). In the model, the receptors are colored in red, the HAMP domains in purple, and the methylation sites (Q297, E304, Q311, E493, E502) are shown as VDW spheres and highlighted in yellow. (C) Overlay between the CSU model and the HAMP-focused refined map. (D-E) The HAMP focused refinement mask (D) and the resulting map (E).

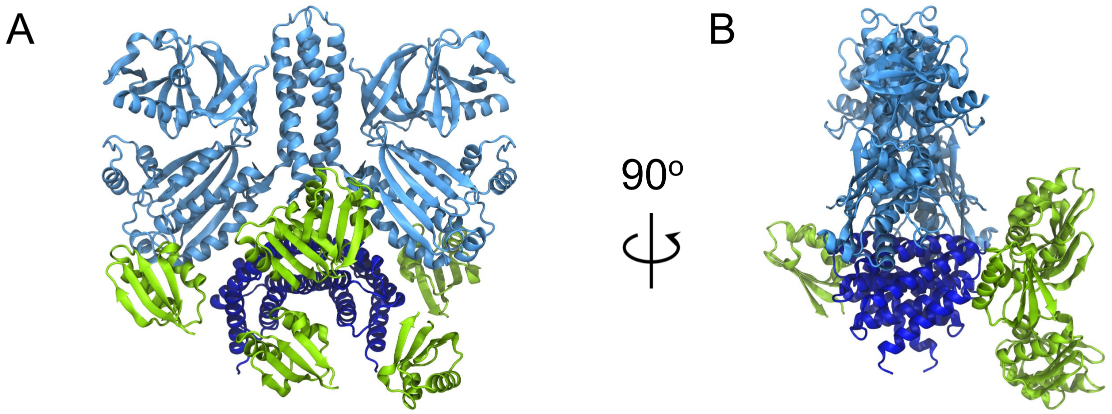

Supplementary Figure 5 | Variable placement of CheA.P2 by AlphaFold2. (A-B) Overlay of five full-length CheA dimer models obtained from Alphafold2, shown from the side (A) and rotated by 90 degrees (B). The position of the P2 domain (green) from one monomer in each model is shown. Note, P2' (not shown) is placed symmetrically with respect to the opposing side of the CheA dimer. For clarity, CheA.P3-P5 (light blue) and CheA.P1 (dark blue), whose positions are unaltered between the models, are shown here for a single model and the disordered linkers connecting P1 to P2 and P2 to P3 are not shown.

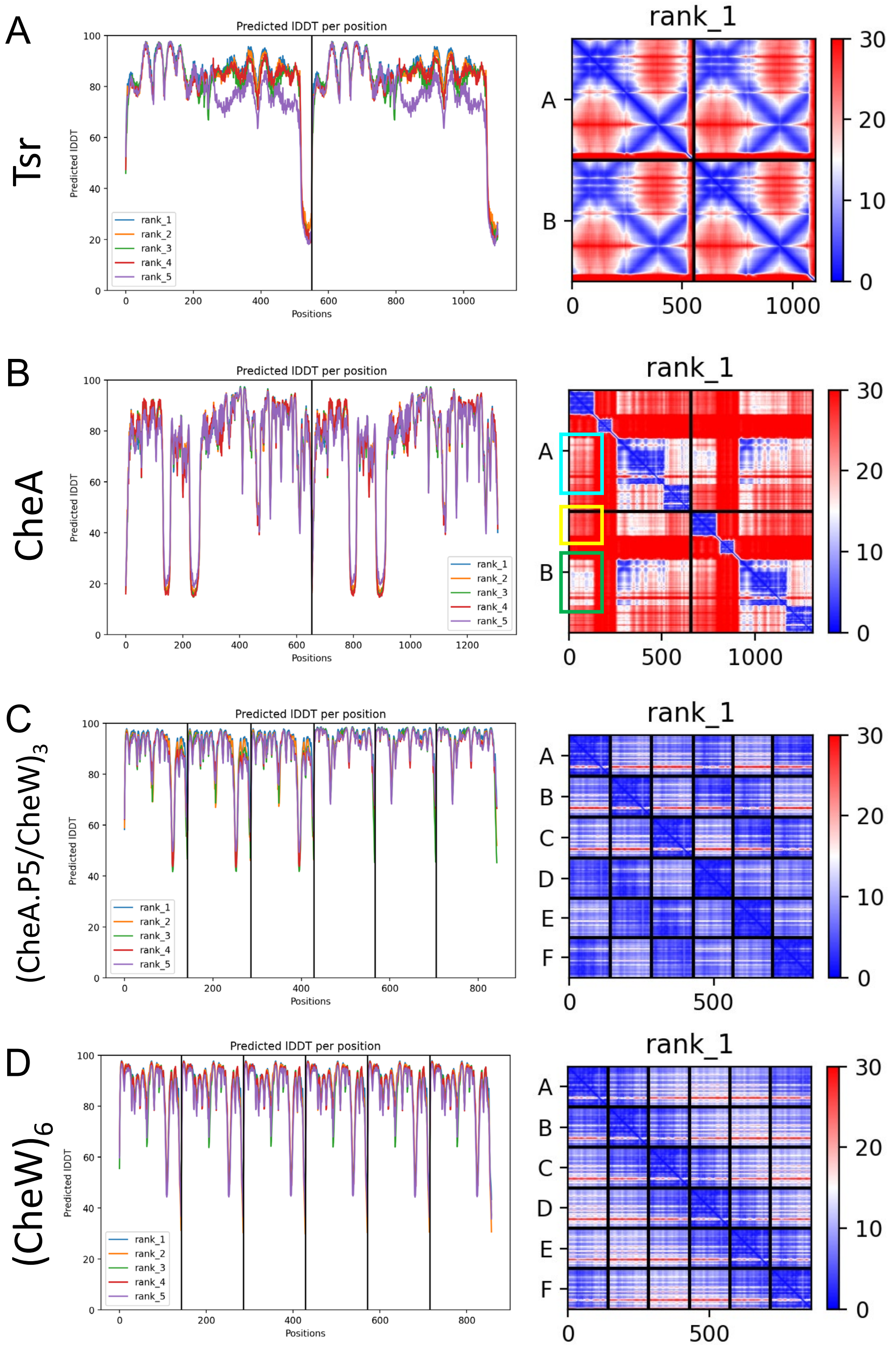

Supplementary Figure 6 | pLDDT and PAE plots for AlphaFold predictions. (A-D) Plots of the Predicted Local Distance Difference Test (pLDDT) and Predicted Aligned Error (PAE) scores output by AlphaFold for full-length Tsr (A), full-length CheA (B), the (CheA.P4/CheW)$_3$ hexameric ring (C), and the (CheW)$_6$ hexameric ring (D). The PAE plot is provided for the top model only. In (B), for one of the P1 domains, PAE regions corresponding to P1/P1, intra-chain P1/P4, and inter-chain P1/P4 interactions are highlighted by yellow, cyan, and green boxes respectively.

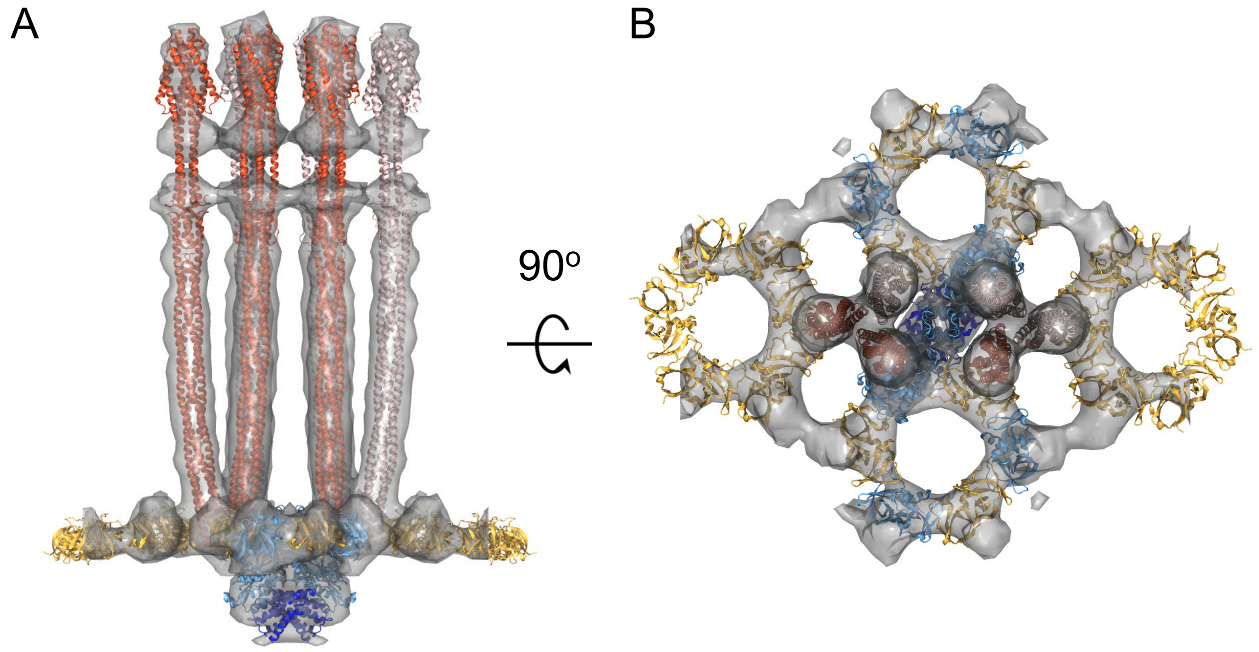

Supplementary Figure 7 | Molecular Dynamics Flexible Fitting (MDFF). (A-B) Overlay between the CSU density map and MDFF-refined CSU model, shown from the side (A) and top (B). The fitted structure included six Tsr homodimers (red/pink), a central CheA.P3-5 dimer (light blue) with associated P1 domains (dark blue), two $(CheA.P5/CheW)_3$ rings (blue and gold), and two $(CheW)_6$ rings (gold).

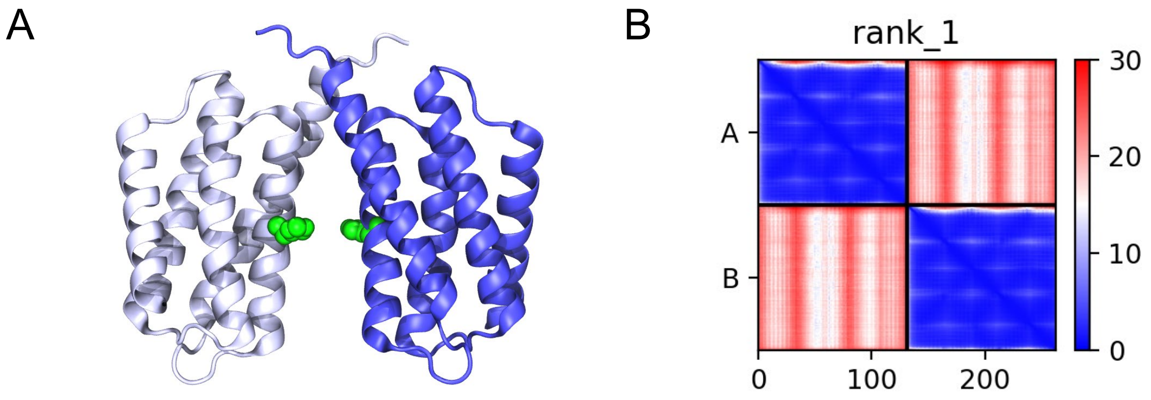

Supplementary Figure 8 | Parallel P1 dimer predicted by AlphaFold for isolated P1 monomers. (A) Coordinates of parallel P1 dimer predicted for isolated *E. coli* P1 domains. P1 monomers are colored in light and dark blue; the substrate histidine is colored green and shown in VDW representation. (B) PAE plot for top model from prediction.

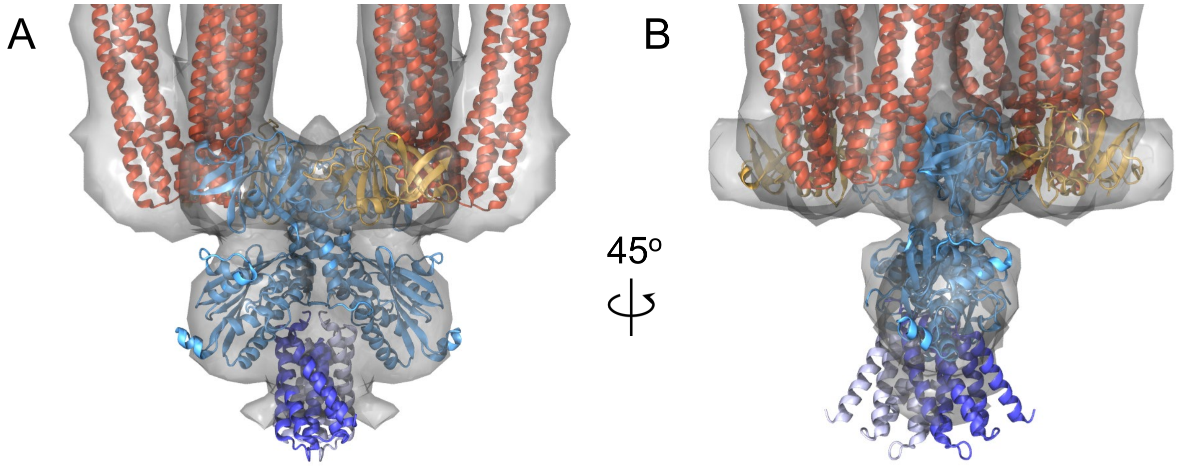

Supplementary Figure 9 | CheA keel density with docked parallel P1 dimer. (A-B) Two views of a parallel P1 dimer model rigidly docked into the excess keel density. Molecules are colored as in other panels.

Movie legends:

Movie 1. Aligned tilt series of an E-gene lyszed native *E. coli* cell displaying a patch of the chemosensory array lattice.

Movie 2. A convolution map resulted from template-matching in emClarity, overlapped with picked subtomograms (green circles).

Movie 3. Extract from MD simulation (50-ns) of CSU model in an atomistic lipid bilayer, illustrating interactions between neighbouring receptor ligand binding domains (LBD). Clusters of residues mediating LBD interactions are shown as VDW spheres and coloured in blue (residues E124, K126, R127, D130) or yellow (residues K99, E102, and K103).
